# Supplementary material for: The Evolution and Characterization of the RNA Interference Pathways in Lophotrochozoa
Source: Genome Biol Evol. 2024 May 7;16(5):evae098. doi: 10.1093/gbe/evae098 (PMC11114477; doi:10.1093/gbe/evae098)
Supplement: evae098_Supplementary_Data [file evae098_supplementary_data.zip › Supplementary_materials.zip!/selected_miRNAs/NoGe_selected_miRNAs/mirdeep_pdf_files/NMRB01000188.1_Notospermus_geniculatus_strain_Ushimado_scaffold188,_whole_genome_shotgun_sequence_6822.pdf]

Provisional ID	:	NMRB01000188.1_Notospermus_geniculatus_strain_Ushimado_scaffold188,_whole_genome_shotgun_sequence_6822
Score total	:	1876.1
Score for star read(s)	:	3.9
Score for read counts	:	1867.6
Score for mfe	:	1.6
Score for randfold	:	0
Score for cons. seed	:	3
Total read count	:	3675
Mature read count	:	3379
Loop read count	:	4
Star read count	:	292

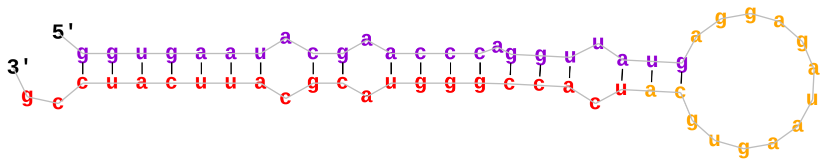

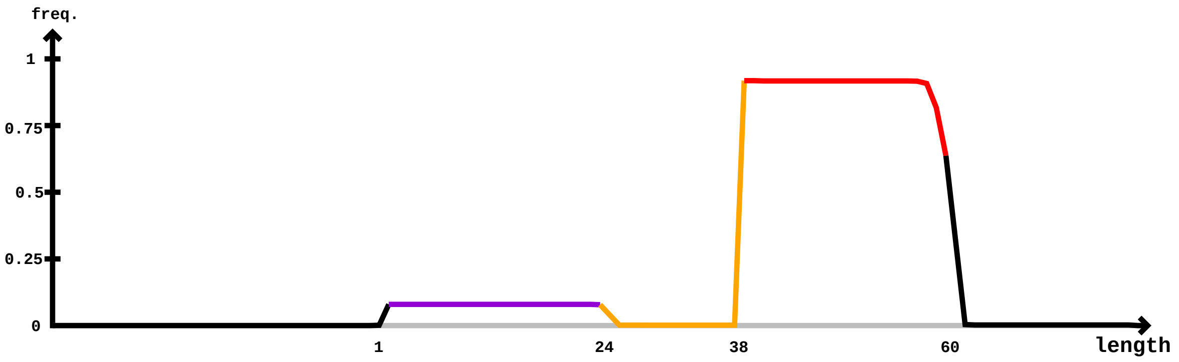


```
                                 Star                                 Mature
5'- ucunuggaauaagaaacaacgcaugucacuuccggugaauacgaacccagguuaugaggagauaagugcaucaccggguacgcauucauccgggugugguggcugugcauuucau   -3'    obs
    ucunuggaauaagaaacaacgcaugucacuuccggugaauacgaacccagguuaugaggagauaagugcaucaccggguacgcauucauccgggugugguggcugugcauuucau          exp
    ............((((...((((.(((((.((((((((((.((.((((.(((.(((............))).))))))).)).))))).))))).)))))))))..))))..   reads       mm          sample
    .................................ggugaauacgaacccagguuaug........................................................   2           0           Mm3
    .................................ggugaauacgaacccaggCuaugagga....................................................   1           1           Mm3
    ......................................................................ucaccggguacgcauucau.......................   2           0           Mm3
    ......................................................................ucaccggguacgcauucauc......................   18          0           Mm3
    ......................................................................ucGccggguacgcauucaucc.....................   1           1           Mm3
    ......................................................................ucaccggguacgcauucaucc.....................   27          0           Mm3
    ......................................................................ucaccggguacgcauuGaucc.....................   1           1           Mm3
    ......................................................................ucaccggguacgcauucaucU.....................   1           1           Mm3
    ......................................................................ucaccggguacgcauucauccU....................   1           1           Mm3
    ......................................................................ucaccggguaUgcauucauccg....................   1           1           Mm3
    ......................................................................ucaccggguacgcauucaucUg....................   1           1           Mm3
    ......................................................................uGaccggguacgcauucauccg....................   1           1           Mm3
    ......................................................................ucaccggguacgcauuAauccg....................   1           1           Mm3
    ......................................................................ucaccggguacgcauucCuccg....................   1           1           Mm3
    ......................................................................ucaccgggCacgcauucauccg....................   1           1           Mm3
    ......................................................................ucaccggguacgcauucauccg....................   120         0           Mm3
    ......................................................................ucaccggguacgcauucauccgU...................   2           1           Mm3
    ......................................................................ucaccggguacgcauucauccgC...................   1           1           Mm3
    ......................................................................ucaccggguaAgcauucauccgg...................   1           1           Mm3
    ......................................................................ucaccggguacgcauucauccgg...................   39          0           Mm3
    ......................................................................ucaccggguacgcauucauccgA...................   1           1           Mm3

    ......................................................ugaggagauaagAgcauc........................................   4           1           Fm3
    ......................................................................ucaccggguacgcauucau.......................   19          0           Fm3
    ......................................................................ucaccggguacgcauucCu.......................   1           1           Fm3
    ......................................................................ucaccggguacgcauucGuc......................   1           1           Fm3
    ......................................................................ucaccggguaUgcauucauc......................   10          1           Fm3
    ......................................................................Acaccggguacgcauucauc......................   1           1           Fm3
    ......................................................................ucaccggguacgcauucauc......................   176         0           Fm3
    ......................................................................ucaccggguacgcauucauU......................   3           1           Fm3
    ......................................................................ucaccggguacgcauuGauc......................   1           1           Fm3
    ......................................................................ucaccggguacgcauucUuc......................   1           1           Fm3
    ......................................................................ucaccggguacgcauuAauc......................   1           1           Fm3
```

```
                                 Star                                 Mature
ucunuggaauaagaaacaacgcaugucacuuccggugaauacgaacccagguuaugaggagauaagugcaucaccggguacgcauucauccggguguggcugugcauuucau
......................................................................ucaGcggguacgcauucauc......................  1     1  Fm3
......................................................................ucaccggguacgcauucaucU.....................  20    1  Fm3
......................................................................ucaccggguacgcauucGucc.....................  1     1  Fm3
......................................................................ucaccggguacgcauucaucA.....................  5     1  Fm3
......................................................................ucaccggguacgcauucCucc.....................  2     1  Fm3
......................................................................ucUccggguacgcauucaucc.....................  1     1  Fm3
......................................................................ucaccggguacgcauucUucc.....................  1     1  Fm3
......................................................................ucaccggguacUcauucaucc.....................  1     1  Fm3
......................................................................ucaccggguacgcauuAaucc.....................  1     1  Fm3
......................................................................ucaccggguacgcauucaucc.....................  345   0  Fm3
......................................................................ucaAcggguacgcauucaucc.....................  1     1  Fm3
......................................................................ucaccggguaGgcauucaucc.....................  1     1  Fm3
......................................................................uAaccggguacgcauucaucc.....................  1     1  Fm3
......................................................................ucaccggguacgcauucauUcg....................  1     1  Fm3
......................................................................ucaccggguacgcauucCuccg....................  1     1  Fm3
......................................................................ucaccggguacgcauuUauccg....................  1     1  Fm3
......................................................................ucaccggguacgcauucauccU....................  4     1  Fm3
......................................................................ucaccggguacgcauucauccg....................  1061  0  Fm3
......................................................................ucaccggguacgcauuGauccg....................  1     1  Fm3
......................................................................ucaccggguaAgcauucauccg....................  3     1  Fm3
......................................................................ucaccgggCacgcauucauccg....................  1     1  Fm3
......................................................................ucUccggguacgcauucauccg....................  2     1  Fm3
......................................................................ucaccggguacUcauucauccg....................  1     1  Fm3
......................................................................ucaccggguacgcauucGuccg....................  4     1  Fm3
......................................................................ucaccggguacgcauuAauccg....................  9     1  Fm3
......................................................................ucaccggguacgcauucauccA....................  5     1  Fm3
......................................................................ucaccggguaGgcauucauccg....................  1     1  Fm3
......................................................................ucaccggguacgcauucUuccg....................  1     1  Fm3
......................................................................ucacUggguacgcauucauccg....................  2     1  Fm3
......................................................................ucCccggguacgcauucauccg....................  1     1  Fm3
......................................................................ucGccggguacgcauucauccg....................  2     1  Fm3
......................................................................ucaccggguaUgcauucauccg....................  2     1  Fm3
......................................................................ucaccgAguacgcauucauccg....................  1     1  Fm3
......................................................................ucaccggguacgcauucaAccg....................  1     1  Fm3
......................................................................ucaccggguacgcauucUuccgg...................  1     1  Fm3
......................................................................ucaccggguaAgcauucauccgg...................  1     1  Fm3
......................................................................ucaccggguacgcauucauccgg...................  208   0  Fm3
......................................................................ucaccggguacgcauucauccgU...................  32    1  Fm3
......................................................................ucaccggguacgcauucauccgA...................  5     1  Fm3
......................................................................ucaccggguacgcauuAauccgg...................  1     1  Fm3
......................................................................uAaccggguacgcauucauccgg...................  1     1  Fm3
......................................................................ucaccggguacgcauucauccggA..................  3     1  Fm3
.............................................................................................guguggcugugcauuuca.  8     0  Fm3

......................................................................ucaccggguacgcauucau.......................  4     0  Mm1
......................................................................ucaccggguacgcauucauc......................  40    0  Mm1
......................................................................ucaccggguaGgcauucauc......................  1     1  Mm1
......................................................................ucaccggguacgcauuUauc......................  1     1  Mm1
......................................................................ucaccgUguacgcauucauc......................  1     1  Mm1
......................................................................ucaccggguacgcauuAauc......................  1     1  Mm1
......................................................................ucaccggguacgcauucaucc.....................  106   0  Mm1
......................................................................ucaccgAguacgcauucaucc.....................  1     1  Mm1
......................................................................ucaccggguacgcauucauccg....................  82    0  Mm1
......................................................................ucaccggguacgcauucGuccg....................  1     1  Mm1
......................................................................ucaccggguacgcauucauccU....................  1     1  Mm1
......................................................................ucaccggguacgcauucauccC....................  8     1  Mm1
......................................................................Ccaccggguacgcauucauccg....................  1     1  Mm1
......................................................................ucaccggguacgcauucUuccg....................  1     1  Mm1
......................................................................ucaccggguacgcauucauccgg...................  13    0  Mm1

.................................ggugaauacgaacccagguuaug........................................................  4     0  Mm2
......................................................................ucaccggguacgcauucau.......................  1     0  Mm2
......................................................................ucaccggguacgcauucauc......................  35    0  Mm2
......................................................................ucaccggguacgcauuUaucc.....................  1     1  Mm2
......................................................................ucaccggguacgcauucaucU.....................  1     1  Mm2
......................................................................ucacUggguacgcauucaucc.....................  1     1  Mm2
......................................................................ucaccggguacgcauucaucc.....................  77    0  Mm2
......................................................................ucaccggguacgcauucGucc.....................  1     1  Mm2
......................................................................ucaccgAguacgcauucaucc.....................  1     1  Mm2
......................................................................ucaccggguacgcauucauccU....................  13    1  Mm2
```

```
                                Star                                  Mature
ucunuggaauaagaaacaacgcaugucacuuccggugaauacgaacccagguuaugaggagauaagugcaucaccggguacgcauucauccggguguggcugugcauuucau
......................................................................ucaccggguacgcauucaucUg....................    1     1   Mm2
......................................................................uUaccggguacgcauucauccg....................    1     1   Mm2
......................................................................ucaccggguacgcauuUauccg....................    1     1   Mm2
......................................................................ucaccggguacgcauucCuccg....................    2     1   Mm2
......................................................................ucaccggguaGgcauucauccg....................    1     1   Mm2
......................................................................ucaccggguCcgcauucauccg....................    1     1   Mm2
......................................................................ucaccggguacgcauucauccg....................  156     0   Mm2
......................................................................ucaccggguacgcauuAauccgg...................    1     1   Mm2
......................................................................ucaccggguacgcauucUuccgg...................    1     1   Mm2
......................................................................ucaccggguacgcauucauccgU...................   14     1   Mm2
......................................................................ucaccggguacgcauucauccgA...................    1     1   Mm2
......................................................................ucaccggguacgcauucauccgg...................   51     0   Mm2
......................................................................ucaccggguacgcauucauccggU..................    1     1   Mm2

.................................ggugaauacgaacccaggCuaug........................................................    1     1   Fm2
.................................ggugaauacgaacccagAuuaug........................................................    2     1   Fm2
.................................ggugaauacgaacccagguuaug........................................................   24     0   Fm2
......................................................................ucaccggguacgcauucaucc.....................   10     0   Fm2
......................................................................ucaccggguacgcauucGuccg....................    1     1   Fm2
......................................................................ucaccggguacgcauucauccg....................   39     0   Fm2
......................................................................ucaccggguacgcauucauccgU...................   10     1   Fm2

................................cggugaauacgaacccagguuau.........................................................    1     0   Fm1
................................cggugaauacgaacccagguuaug........................................................    3     0   Fm1
.................................ggugaauacgaacccagguua..........................................................    4     0   Fm1
.................................ggugaauacgaacccagguuau.........................................................    4     0   Fm1
.................................ggugaauacgaacUcagguuaug........................................................    1     1   Fm1
.................................ggugaauacgaacccagguuaug........................................................  192     0   Fm1
.................................ggugaauacgaacccagguuauA........................................................    5     1   Fm1
.................................ggugaauacgaacccagUuuaug........................................................    4     1   Fm1
.................................ggugaauacgaacccagCuuaug........................................................    1     1   Fm1
.................................ggugaauacgaacccagAuuaug........................................................    3     1   Fm1
.................................ggugaauacgaacccagguuaugU.......................................................   40     1   Fm1
.................................ggugaauacgaacccagguuaugaggagauaagugca..........................................    1     0   Fm1
......................................................................ucaccggguacgcauucU........................    3     1   Fm1
......................................................................ucaccggguacgcauucau.......................    4     0   Fm1
......................................................................ucaccggguacgcauucUu.......................    1     1   Fm1
......................................................................ucaccggguacgcauucauc......................   40     0   Fm1
......................................................................ucaccggguacgcauucauU......................    2     1   Fm1
......................................................................ucaccggguacgcauucUucc.....................    1     1   Fm1
......................................................................ucaccggguacgcauucaucU.....................    1     1   Fm1
......................................................................ucaccggguacgcauucaucA.....................    1     1   Fm1
......................................................................ucaccggguacgcauucaucc.....................   52     0   Fm1
......................................................................ucaccggguacgcauucUuccg....................    1     1   Fm1
......................................................................ucaccggguaAgcauucauccg....................    1     1   Fm1
......................................................................ucaccggguacgcauuAauccg....................    2     1   Fm1
......................................................................ucaccggguacgcauucauccg....................  190     0   Fm1
......................................................................uAaccggguacgcauucauccg....................    1     1   Fm1
......................................................................ucaccggguacgcauucGuccg....................    1     1   Fm1
......................................................................ucaccggguacgcauucauccgg...................  189     0   Fm1
......................................................................ucaccggguacgcauucauccgU...................   24     1   Fm1
......................................................................ucaccggguacgcauucauccgA...................    1     1   Fm1
......................................................................ucaccCgguacgcauucauccgg...................    1     1   Fm1
......................................................................ucaccggguacgcauucUuccgg...................    1     1   Fm1
......................................................................ucaccggguacgcauucCuccgg...................    1     1   Fm1
......................................................................ucaccggguaGgcauucauccgg...................    1     1   Fm1
......................................................................ucaccggguacgcauucauccggA..................    2     1   Fm1
```
